# Sustainability of public health interventions: where are the gaps?

**DOI:** 10.1186/s12961-018-0405-y

**Published:** 2019-01-15

**Authors:** David Roger Walugembe, Shannon Sibbald, Marlene Janzen Le Ber, Anita Kothari

**Affiliations:** 1 School of Health Studies, Arthur and Sonia Labatt Health Sciences Building, Rm. 222, London, Ontario N6A 5B9 Canada; 2 Faculty of Information and Media Studies, The University of Western Ontario, FIMS & Nursing Building, Room 2050, London, ON N6A 5B9 Canada; 3 Department of Family Medicine, Schulich School of Medicine & Dentistry, Western University, 1465 Richmond St, London, ON N6G 2M1 Canada; 4 School of Leadership and Social Change, Brescia University College, 1285 Western Road, London, Ontario N6G 1H2 Canada

**Keywords:** Sustainability, public health, interventions, programmes

## Abstract

The current scholarly focus on implementation science is meant to ensure that public health interventions are effectively embedded in their settings. Part of this conversation includes understanding how to support the sustainability of beneficial interventions so that limited resources are maximised, long-term public health outcomes are realised, community support is not lost, and ethical research standards are maintained. However, the concept of sustainability is confusing because of variations in terminology and a lack of agreed upon measurement frameworks, as well as methodological challenges. This commentary explores the challenges around the sustainability of public health interventions, with particular attention to definitions and frameworks like Normalization Process Theory and the Dynamic Sustainability Framework. We propose one important recommendation to direct attention to the sustainability of public health interventions, that is, the use of theoretically informed approaches to guide the design, development, implementation, evaluation and sustainability of public health interventions.

## Introduction

There is increasing interest and effort among researchers, evaluators, funders and community partners to understand the sustainability of effective public health interventions [1]. Despite these efforts, understanding sustainability is hindered by several challenges such as variations in conceptualising sustainability, lack of agreed upon measurement frameworks, difficult reporting approaches and timing, as well as methodological challenges related to sustainability. The purpose of this paper is to explore the challenges around the sustainability of public health interventions and discuss how to address them. Failing to do so can result in wasted funds [2] and missed opportunities to improve the population’s health and wellbeing.

First, we consider the definitions of sustainability followed by a discussion of the importance of sustainability. We then examine some of the theories, models and frameworks that have been proposed to guide the evaluation of sustainability of public health interventions. To do this, we explored relevant literature through online database searches using the words ‘public health’, ‘sustainability’, ‘health policies’, ‘integration’ and ‘implementation’. The PubMed, CINHAL, Scopus and Google Scholar databases were searched for articles exploring sustainability and how it relates to public health. We conclude with a key recommendation to improve sustainability of public health interventions, using theoretically informed approaches to guide the design, development, implementation, evaluation and sustainability of public health interventions.

### Defining sustainability

Despite the increase in the health literature discussing sustainability, there remains little consensus as to its definition. Terms such as ‘continuation’, ‘maintenance’, ‘durability’, ‘adoption’, ‘embedding’, ‘incorporation’, ‘appropriation’, ‘continuance’, ‘integration’, ‘institutionalisation’, ‘routinisation’, ‘long term’ and ‘adherence’, among others, have been used to describe sustainability [3–6]. Shediac-Rizkallah and Bone [3] conceptualise sustainability broadly as “*the maintenance of health benefits over time*”, whereas the United States Agency for International Development (USAID) defines sustainability through a more focused definition of “*the capacity to maintain program services at a level that will provide ongoing prevention and treatment for a health problem after termination of major financial, managerial and technical assistance from an external donor*” [6]. Scheirer and Dearing [7] focus exclusively on programme elements and define sustainability as “*the continued use of program components and activities for the continued achievement of desirable program and population outcomes*”. This definition has been adapted by Schell et al. [1], who consider the element of time and thus define sustainability as “*the ability to maintain programming and its benefits over time*”.

The above definitions have nuanced differences in the conceptualisation of sustainability and each of them highlights distinct, yet interrelated constructs of sustainability. As noted by Moore et al. [8], the above definitions are based upon three key main constructs presented by Scheirer and Dearing [7] and are derived from a framework by Shediac-Rizkallah and Bone [3]. These constructs include continuation of health benefits for individuals after initial programme funding ends, continuation of programme activities within one or more organisations, and building of a community’s capacity to develop and deliver programmes [8]. This commentary adopts the definition of sustainability advanced by Scheirer and Dearing [7].

Noting the multiplicity of the terminologies and constructs across definitions of sustainability, Moore et al. [8] systematically developed a comprehensive definition of sustainability as gleaned from existing literature. They defined sustainability based on five common constructs that describe individual and organisational sustainability, including time, continued delivery, behaviour change, evolution/adaptation and continued benefits. These efforts towards a comprehensive definition of sustainability have been augmented by Lennox et al. [9], whose review of existing literature identified five distinct definitions of sustainability listed in Table 1 below.

**Table 1:** Comparisons of sustainability definitions

Sustainability factors compared among frameworks	Definition constructs – Lennox et al. [9]	Description – Lennox et al. [9]	Definition constructs – Moore et al. [8]	Description – Moore et al. [8]
Core activity	Continued programme activities	The ability of activities to continue, appropriate to the local context, after withdrawal of external funding	Continued delivery or institutionalisation of a clinical intervention or programme	Whether an organisation or community is continuing to provide a programme (such as delivering multidimensional treatment foster care) or continuing to use the strategies necessary to support behaviour change (e.g. education, audit and feedback)
Recipient benefits	Continued health benefits	The ability to sustain population health outcomes	Continued health benefits for individuals/systems	Maintenance of outcomes after initial implementation phase
Implementation	Not included		Maintenance of behaviour change at individual clinician/patient level	Whether the implementer is following the recommendations of the evidence-based programme, guideline, or practice (how the implementer is interacting with patients, clients or community members)
Adaptation	Evolution/Adaptation	Adapting successfully to change and providing a range of valued service delivery opportunities and practices in an effective and efficient manner	Evolution/Adaptation	Either changes in the programme or implementation strategies or changes in individual’s maintenance of behaviour
Organisational value	Capacity-building	Relates to inter-organisational relationships that might serve as the basis of collaborative problem-solving capacity	Not included	
Cost-benefit	Recovering costs	The ability of an organisation to produce outputs of sufficient value so that it acquires enough inputs to continue production at a steady or growing rate	Not included	
Longitudinal PErspective	Not included		Time	Not elaborately described although assertions such as “*after initial funding ends*” and “*over time*” are used to denote time

Despite these efforts, the approaches to sustainability, articulation and specific outcomes of interest addressed by the above constructs continue to vary. No sustainability approach contains a similar combination of constructs and nor do any of them capture all the identified constructs (refer to Table 1 below). For example, efforts by Lennox et al. [9] have identified 40 constructs grouped under six emergent themes, including general resources, demonstrating effectiveness, monitoring progress over time, stakeholder participation, integration with existing programmes and policies, training and capacity-building; these are highlighted in the ‘Consolidated framework for sustainability constructs in healthcare’ in Table 2 below. Thus, further efforts towards a workable and feasible definition of sustainability are still required.

**Table 2:** Consolidated framework for sustainability constructs in healthcare (adapted from Lennox et al. [9])

The initiative design	Negotiating initiative processes	The people involved	Resources	The organisational setting	The external environment
Demonstrating effectiveness	Belief in the initiative	Stakeholder participation	General resources	Integrating with existing programmes and policies	Socioeconomic and political considerations
Monitoring progress over time	Accountability of roles and responsibilities	Leadership and champions	Funding	Intervention adaptation and receptivity	Awareness and raising the profile
Training and capacity-building	Defining aims and shared vision	Relationships and collaboration and networks	Infrastructure	Organisational values and culture	Urgency
Evidence base for the initiative	Incentives	Community participation	Resources – staff	Organisational readiness and capacity	Spread to other organisations
Expertise	Workload	Staff involvement	Resources – time	Support available	
The problem	Complexity	Ownership		Opposition	
Project duration	Job requirements	Power			
Improvement methods		Patient involvement			
Project type		Satisfaction			

### Importance of sustainability

The increasing appreciation for the importance of sustainability is partly due to a recognition by researchers and funders that, without programme sustainability, studying implementation of interventions is very challenging [10]. There are four main reasons cited as to why sustainability should concern public health practitioners [3, 4]. First, sustained programmes’ long-term effects are easier to study since they are often maintained over a longer period. For example, the effects of behaviour change interventions, such as smoking cessation, are only seen several years after the intervention has been implemented [11]. Second, often, changes in community health are not detectable until 3–10 years after the beginning of programmes; this is referred to as the latency period between the beginning of the programme-related activities and their effects on population health [12]. Third, a focus on sustainability requires us to consider potential loss of investments for organisations and people in the event that interventions with perceived or actual benefits are not sustained. Many interventions require substantial investment in terms of human, fiscal and technical resources; inadequate attention to the sustainability of such investments could be considered careless and inefficacious. Lastly, discontinuation of interventions without planning or intention causes disillusionment of participants and poses obstacles to future community mobilisation efforts [3, 4]. For example, new interventions may encounter diminished community support and trust in communities with a history of interventions that were abruptly or inappropriately terminated [3].

While advancing the “*conceptual framework for program sustainability in public health*”, Schell et al. [1] note that programmes that are able to sustain themselves are more likely to produce lasting outcomes and result in healthier outcomes. Maximising the benefits accrued from studying the sustainability of evidenced-informed interventions is an essential task for researchers and programme planners.

There are also ethical concerns related to sustainability of interventions within the broader context of translational and dissemination research [7]. Researchers who use an ethical lens consider whether it is ethical to collect data from communities, build and implement interventions, and then abandon the communities when funding ends or when the intervention does not achieve the intended benefits for research purposes [7]. In a similar way, funders who use an ethical lens consider the implications as to whether it is ethical to develop interventions without adequate support to sustain the programmes if they prove effective [7]. These concerns warrant developing a further understanding of and guidance for sustainability in public health interventions.

### Sustainability: theories, models and frameworks

There have been several theories proposed to guide the evaluation of sustainability of public health interventions. A recent paper by Luke et al. [13] found 17 frameworks for sustainability in the public health literature; however, there are only two tools that actually measure sustainability. The Health TAPESTRY Implementation Guide lists five frameworks and tools [14], and there are also theories that consider sustainability such as the Normalization Process Theory (NPT) [15] and the Dynamic Sustainability Framework (DSF) [10].

NPT provides “*a set of sociological tools that facilitate understanding and explanation of the social processes through which new or modified practices of thinking, enacting and organizing work are operationalized in healthcare and other institutional settings*” [16]. As a sociological theory, NPT goes beyond implementation and examines embedding and integration of embedded practices in their social contexts. May and Finch define ‘embedding’ as making practices routine elements of everyday life (normalising) and ‘integration’ as “*the sustaining of the embedded practices in their social contexts*”. NPT recognises and addresses the concomitant nature of implementation and sustainability of new interventions [4, 16]. NPT has been used to understand the implementation and embedding of several social processes, including public health interventions. For example, Hooker et al. [17] used NPT to evaluate the implementation of a best practice model of family violence screening; the use of NPT assisted in explaining what aspects of the intervention worked and what did not, as well as identifying processes that were likely to enhance sustainability. Further, they noted that the use of maternal health and well-being checklists facilitated normalisation/integration of the maternal and child health nurse screening and care for mothers experiencing domestic violence intervention [17]. By using NPT, the authors were able to conclude that the use of checklists facilitated cognitive participation and collective action, specifically interactional workability between the clinicians and clients [17]. Both parties embraced the checklists, which in turn allowed for increased dialogue, trust and self-completion. The use of NPT further enabled Hooker et al. [17] to identify detrimental aspects to normalisation of interventions, including excessive workloads with rigid routines and reduced capacity for networking and appraisal. Based on their study findings, the authors recommend paying attention to organisational support (contextual integration), and note that recording and monitoring work (reflexive monitoring) are essential for sustainability of a practice.

The DSF also attempts to address sustainability. The framework focuses on the intervention, the context and the broader ecological system in which the practice settings operate. The DSF emphasises that change exists – in the use of interventions over time, the characteristics of practice settings and the characteristics of the broader system that establishes the context for how care is delivered. What distinguishes the DSF from other implementation frameworks is its consideration of these elements (i.e. intervention, context and the broader ecological system) over time [10]. By considering time, the DSF framework acknowledges the dynamism and complexity within the health system and in the context. It does not assume a static service delivery system in which sustainability can only be assessed at key points in time. However, reports regarding the utility of the DSF framework in studying the sustainability of public health interventions still remain limited. Although Klinga et al. [18] reportedly used DSF in describing factors that contribute to the achievement of sustainable integration of health and social care for persons with complex needs in Sweden, details of lessons to be gleaned from this study are yet to be published.

A number of reasons have been advanced to explain the limited focus on the sustainability of public health interventions. Key among these is the temporal aspect of sustainability. Sustainability is often conceptualised as a final phase of programme development after planning, implementation and evaluation phases [4]. As a result of this conceptualisation, models such as RE-AIM (Reach Effectiveness, Adoption, Implementation and Maintenance) [19] and PRECEDE-PROCEED (Predisposing, Reinforcing and Enabling Constructs in Educational Diagnosis and Evaluation- Policy, Regulatory and Organizational Constructs in Education and Environmental Development) [20, 21] do not adequately consider the importance of sustainability in designing interventions [22]. This in turn potentially limits the likelihood of sustainability of interventions. Pluye et al. [4] argue that programme implementation and sustainability are parallel and concomitant processes; they advocate for the re-conceptualisation of sustainability to ensure that it is integrated into the design of interventions and planned for in advance.

Limited reporting on the use of theories and their impacts in studying the implementation of public health interventions further accounts for the limited understanding and appreciation of sustainability-focused theories, models and frameworks. Glanz and Bishop [21] note that the limited reporting of theory use in the implementation of interventions could be attributed to the limited understanding of how to measure and analyse constructs of various theories. Despite drawing from a variety of fields and disciplines, including psychology, sociology, social psychology, anthropology, communications, nursing, marketing, management sciences, and education, among others [21–23], very few of the existing public health implementation theories and models explicitly report on sustainability. Colquhoun et al. [23] note that most theories are used for the justification, conceptualisation and development of the intervention as well as the prediction and discussion of results, but sustainability is less often considered. It is therefore important to explore the use of theories and their impacts in studying the sustainability of public health interventions [3, 5, 21, 22, 24].

### Sustainable public health

There is increasing concern and importance accorded to sustainability of public health interventions. For example, through a comprehensive review of literature and concept mapping processes, Schell et al. [1] developed a nine-domain conceptual framework for programme sustainability capacity for public health. The identified domains of capacity for sustainability framework include political support, funding stability, partnerships, organisational capacity, programme evaluation, programme adaptation, communication, public health impacts and strategic planning. The purpose of this framework is to help create a shared understanding of sustainability across a variety of public health decision programme stakeholders such as decision-makers, practitioners, funders, researchers and evaluators [1]. Like Chambers et al. [10], Schell et al. [1] observe that sustainability of interventions and their benefits is determined by interrelations among the identified domains over a period of time. They further advocate for the continued assessment of the characteristics of the intervention, its parent (host) organisation, and the larger service system context in which both the intervention and organisation operate [1].

In an attempt to address the conceptualisation and evaluation of successful implementation efforts, Proctor et al. [25] advanced a taxonomy of “*implementation outcomes*” which endeavours to address sustainability. The taxonomy proposes eight conceptually distinct outcomes of an evaluation, including acceptability, adoption/uptake, appropriateness, costs, feasibility, fidelity, penetration and sustainability [25]. According to this categorisation, sustainability is defined as “*the extent to which a newly implemented intervention is maintained or institutionalised within a service setting’s ongoing stable operations*” [25]. This definition of sustainability emphasises the institutionalisation of sustainability within an organisation. Rabin et al. [26] observe that three stages determine institutionalisation of an intervention. These include single events such as the transition from temporary to permanent funding (passage), repeated reinforcement of the intervention through including it in organisational or community procedures and behaviours (cycle routine), and the extent to which the intervention is integrated into all the subsystems of an organisation (niche saturation) [26].

Despite the increasing concern and importance accorded to sustainability of public health interventions, most of the efforts remain at a conceptual stage with sustainability and related concepts appearing more often in conceptual papers than in empirical articles. Additionally, there is a shortage of tools to measure the sustainability of interventions [25].

### Improving sustainability in public health

We have one key recommendation to improve sustainability of public health interventions, namely the use of theoretically informed approaches to guide the design, development, implementation, evaluation and sustainability of public health interventions.

Proponents for the use of theory to explore sustainability argue that it facilitates the understanding of various influences on the implementation of complex interventions, including the contexts, characteristics of the target audiences and the intervention itself [15, 16, 22, 23, 27–29]. Others argue that using theoretically informed approaches provides better understanding as to why some interventions fail and some apparently similar approaches succeed [21, 23, 27, 28]. Furthermore, it has been observed that generalising through theory potentially offers a more efficient and appropriate method of generalisation than study replication in varied study settings. The ability to generalise through theory facilitates acquisition of information by decision-makers on how effective interventions are scaled up from small trials into broader policy and practice [27, 28, 30–32]. Therefore, to improve sustainability of public health interventions we recommend the increased use of theoretically informed approaches such as NPT and DSF to design, develop, implement, evaluate and sustain interventions.

## Conclusion

This commentary adopts the definition by Scheirer and Dearing [7], who acknowledge a more fulsome conceptualisation and believe that, despite the nuanced differences in the sustainability terminologies, the core understanding of sustainability includes the continued use of programme components and activities beyond their initial funding period and even to their desired intended outcomes. The purpose of this paper is to explore the challenges around the sustainability of public health interventions and discuss how to address them. Amidst the challenges of and limited emphasis on the sustainability of interventions across several public health implementation theories, frameworks and models, the increased interest among researchers, evaluators, funders and community partners to improve the understanding of sustainability calls to invest resources and research in this area. To improve sustainability of public health interventions, we suggest prioritising the usage of theoretically informed approaches such as NPT and DSF to design, develop, implement, evaluate and sustain interventions. We acknowledge that this is not a simple recommendation but believe that, as more work is done under this guidance, public health programmes will succeed, sustain and ultimately benefit more people. The goal of any public health intervention should include a consideration for sustainability; we hope we furthered this point and provided a step toward achieving it.

An important next step is to conduct a more thorough and comprehensive review of literature, listing all the different sustainability frameworks, models and theories by their name, source, constructs and validation status. This review will also attempt to classify how these are used, for example, as frameworks versus tools. It will document how various sustainability frameworks, models and theories are applied across health-related fields and programmes, for example, in public health versus environmental health or smoking cessation versus clinical well-child visits. Finally, the review will also attempt to document the settings in which the different sustainability frameworks, models and theories are used such as in low-income versus high-income settings.

## Abbreviations

DSF: Dynamic Sustainability Framework
NPT: Normalization Process Theory
